# Phosphatidylethanolamine dynamics are required for osteoclast fusion

**DOI:** 10.1038/srep46715

**Published:** 2017-04-24

**Authors:** Atsushi Irie, Kei Yamamoto, Yoshimi Miki, Makoto Murakami

**Affiliations:** 1 Lipid Metabolism Project, Tokyo Metropolitan Institute of Medical Science, Setagaya-ku, Tokyo, 156-8506, Japan; 2 Faculity of Bioscience and Bioindustry, Tokushima University, Tokushima, 770-8513, Japan; 3 PRIME, Japan Agency for Medical Research and Development, Chiyoda-ku, Tokyo 100-0004, Japan; 4 AMED-CREST, Japan Agency for Medical Research and Development, Chiyoda-ku, Tokyo 100-0004, Japan

## Abstract

Osteoclasts, responsible for bone resorption, are multinucleated cells formed by cell-cell fusion of mononuclear pre-osteoclasts. Although osteoclast fusion is a pivotal step for osteoclastogenesis, little is known about the mechanism involved. To clarify the underlying process, we investigated dynamics of membrane phospholipids during osteoclastogenesis *in vitro.* We found that the cellular content of phospholipids, phosphatidylethanolamine (PE) in particular, was increased during osteoclast differentiation. Furthermore, PE was greatly increased in the outer leaflet of the plasma membrane bilayer during osteoclastogenesis, being concentrated in filopodia involved in cell-cell fusion. Immobilisation of the cell surface PE blocked osteoclast fusion, revealing the importance of PE abundance and distribution. To identify the molecules responsible for these PE dynamics, we screened a wide array of lipid-related genes by quantitative PCR and shRNA-mediated knockdown. Among them, a PE-biosynthetic enzyme, acyl-CoA:lysophosphatidylethanolamine acyltransferase 2 (LPEAT2), and two ATP-binding cassette (ABC) transporters, ABCB4 and ABCG1, were markedly increased during osteoclastogenesis, and their knockdown in pre-osteoclasts led to reduction in PE exposure on the cell surface and subsequent osteoclast fusion. These findings demonstrate that the PE dynamics play an essential role in osteoclast fusion, in which LPEAT2, ABCB4 and ABCG1 are key players for PE biosynthesis and redistribution.

Bone metabolism involves a continuous process of remodelling that is maintained as a strictly coupled balance between bone formation by osteoblasts and bone resorption by osteoclasts. Osteoclasts are multinucleated cells derived from hematopoietic precursors of the monocyte/macrophage lineage. The hematopoietic precursors differentiate into mononucleated pre-osteoclasts upon stimulation with macrophage colony-stimulating factor (M-CSF) and receptor activator of nuclear factor-κB ligand (RANKL), which are essential for osteoclast differentiation^1^. At a late stage of osteoclast differentiation, mononucleated pre-osteoclasts undergo cell-cell fusion with one another to form multinucleated mature osteoclasts.

Recent studies have identified particular molecules that are involved in osteoclast fusion. Dendritic cell-specific transmembrane protein (DC-STAMP) is required for multinucleation of osteoclasts^2^. In addition, vacuolar (H^+^) ATPase (v-ATPase), E-cadherin, a disintegrin and metalloproteinase domain-containing protein (ADAM)8, ADAM12, CD9, CD44, CD47 and osteoclast stimulatory transmembrane protein have also been implicated in osteoclast fusion^3^. However, the molecular mechanisms whereby these molecules facilitate osteoclast fusion are still obscure.

Cell-cell fusion is a process critical for development and maintenance of several tissues, including fertilisation, myoblast fusion, macrophage giant cell formation and osteoclastogenesis^4^. The process of cell fusion consists of sequential steps: First, cells recognise their fusing partner cells, and then phospholipid bilayers in the cellular plasma membrane come into contact with each other, followed by formation of a hemifusion intermediate structure in which the outer leaflets of apposed membranes merge while the inner leaflets remain distinct. This intermediate conformation then breaks and a fusion pore is formed, the latter finally expanding to complete the fusion process^5^. Thus, during cell-cell fusion, the structure of the lipid bilayers undergoes dynamic change, whereby the stability and fluidity of the bilayers are altered.

Phospholipids are the most fundamental components of the lipid bilayer in cell membranes. They are classified into several groups based on their hydrophilic head groups, which include phosphatidylcholine (PC), phosphatidylethanolamine (PE), phosphatidylserine (PS), phosphatidylinositol (PI) and sphingomyelin (SM). Phospholipids can be biosynthesised via different pathways: the major route for phospholipid biosynthesis is a *de novo* pathway, in which the head groups of phospholipids are transferred to diacylglycerol^6^. Phospholipids are also remodelled through rapid turnover of fatty acyl chains through deacylation by phospholipases and reacylation by acyl-CoA-dependent lysophospholipid acyltransferases (LPLATs)^7^,^8^. In addition, PE is synthesised through decarboxylation of PS catalysed by PS decarboxylase^9^. Although the phospholipid compositions and distributions in biological membranes vary among animal species, cell types and intracellular organelles, phospholipids are distributed asymmetrically in the plasma membrane of most mammalian cells; PC/SM and PE/PS/PI are localised in the outer and inner leaflets, respectively^10^. Three functional groups of lipid transporters distributed across the outer and the inner leaflets of the lipid bilayer regulate this asymmetric distribution of phospholipids. Flippases, which include the P-type ATPase superfamily, constitute the first group, transporting lipids from the outer to the inner leaflet^11^. Floppases, constituting the second group, transport lipids from the inner to the outer leaflet, and this activity is attributed to ATP-binding cassette (ABC) transporters^11^. The third group of lipid transporters, called scramblases, mobilises lipids bidirectionally between the outer and inner leaflets, and include the anoctamin (also known as transmembrane protein (TMEM) 16) and Xk-related (Xkr) protein families^12^.

Since the head group of PE is smaller than those of other phospholipids, PE prefers the concave volume of the inner leaflet of the lipid bilayer^13^, and the localisation of PE in the inner plasma membrane stabilises the morphology of cells. However, PE translocates to the outer leaflet at the cleavage furrow when cells divide^14^. Moreover, biophysical studies have shown that PE-rich liposomes promote formation of the hemifusion intermediate structure during membrane fusion^15^. These observations suggest that PE is dynamically redistributed within the lipid bilayer, leading to changes in membrane curvature and thereby the morphology of cells. In addition to the head groups, the hydrophobic fatty acid tails of phospholipids also affect the characteristics of membranes. Unsaturated fatty acids have kinked carbohydrate chains, which interfere with the highly ordered packing of membranes, and therefore phospholipids containing unsaturated fatty acids reduce the stability of the membrane and increase its fluidity^16^.

Although phospholipids are the principal components of cellular membranes and are expected to be key players in cell-cell fusion, their compositions and distributions during the process of cell fusion, including that in osteoclastogenesis, have been poorly characterised. Here we have investigated the dynamics of phospholipids during osteoclastogenesis and provide the first evidence that redistribution of PE in the plasma membrane is essential for osteoclast fusion.

## Results

### Increase of the proportion of PE during osteoclastogenesis

**Figure 1:**
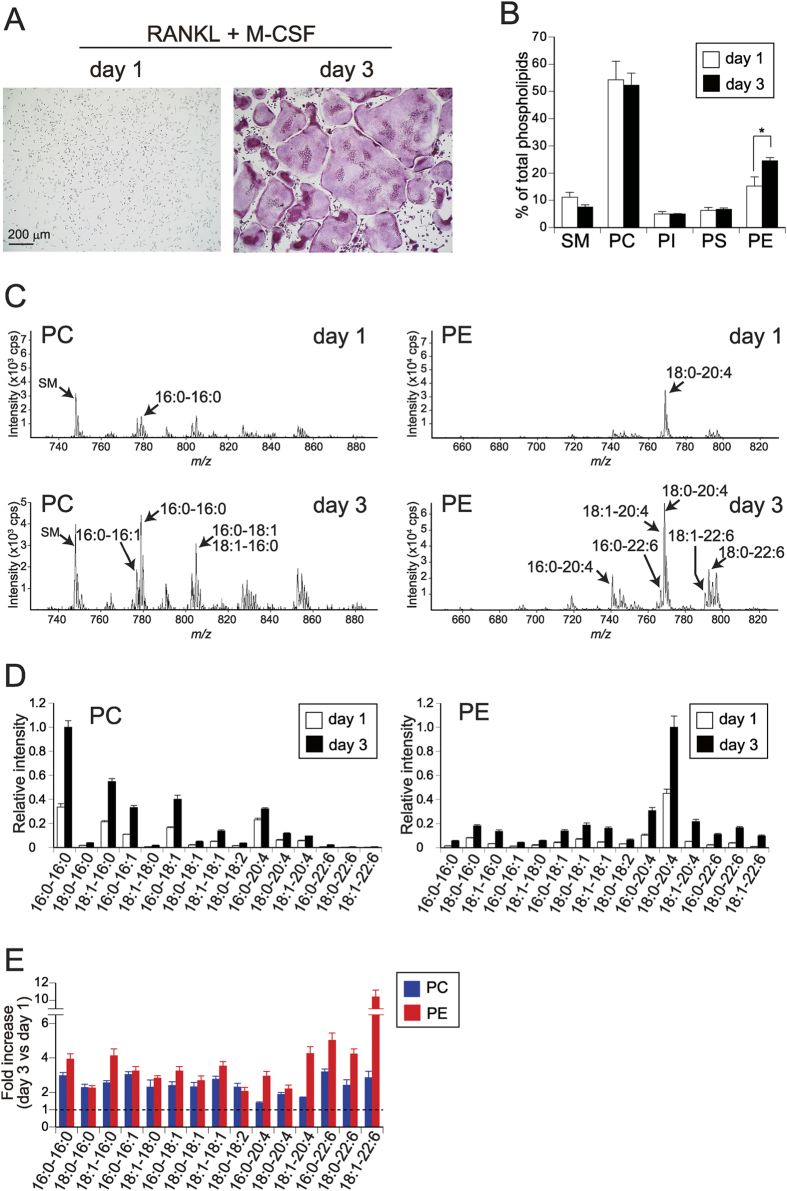
Increase of PE content during osteoclastogenesis. (**A**) Osteoclast differentiation *in vitro*. Bone marrow cells were cultured with M-CSF and TGF-β for 3 days (osteoclast precursors) and then for an additional 1 day (immature osteoclasts) or 3 days (mature osteoclasts) with RANKL and M-CSF. The cells were stained for TRAP. (**B**) Phospholipid compositions of immature and mature osteoclasts. Osteoclast precursors (6 × 10^5^ cells/well in 6-well plates) were cultured for 1 or 3 days with RANKL and M-CSF, and lipids extracted from these cells were separated by two-dimensional thin layer chromatography and quantified. The percentages of individual phospholipid species relative to total phospholipids are shown (n = 6). (**C**) Representative MS profiles of PC and PE species in immature and mature osteoclasts. Phospholipids extracted from the cells cultured in a 96-well plate were applied to the MS analysis. Peaks for major molecular species are indicated. (**D**) Quantitative results of MS analysis (n = 4). (**E**) Fold increase (day 3 versus day 1) of the individual phospholipid species quantified in (**D**) (n = 4). A dashed line (fold increase of 1) indicates that the amounts before and after differentiation are equivalent. Each value is the mean ± SE.

Osteoclast precursors were generated from mouse bone marrow cells by culture with M-CSF and transforming growth factor (TGF)-β and differentiated into multinucleated mature osteoclasts expressing tartrate-resistant acid phosphatase (TRAP), a marker enzyme for osteoclast differentiation^17^, by culture with RANKL and M-CSF *in vitro*. Accordingly, although no TRAP-positive multinucleated cells were observed after treatment with RANKL and M-CSF for 1 day (Fig. 1A), TRAP-positive multinucleated cells began to appear after culture with RANKL and M-CSF for 2 days, and most of these cells became fused, giving rise to multinucleated osteoclasts, on days 3–4 (Fig. 1A). To investigate phospholipids during osteoclastogenesis, we extracted them from undifferentiated and differentiated osteoclasts and analysed their compositions. Total phospholipid contents in the cells were 11.21 ± 1.63 and 50.60 ± 5.58 nmol/well on day 1 and day 3, respectively, revealing an approximately 4~5-fold increase after osteoclast differentiation and fusion. Although the compositions of PC, PI and PS relative to total phospholipids were not significantly changed, the ratio of PE in differentiated cells was significantly higher than that in undifferentiated cells, accompanied by a trend toward reciprocal reduction of the ratio of SM (Fig. 1B).

To further assess the alterations of phospholipids, we performed mass spectrometry (MS)-based lipidomics using undifferentiated and differentiated osteoclasts. Representative MS patterns of total PC and PE extracted from cells cultured in a 96-well plate are shown in Fig. 1C, where most PC and PE species were increased after differentiation. A notable difference between these two phospholipids was that saturated and monounsaturated fatty acids were abundant in the *sn*-2 position of PC, whereas that of PE was enriched in polyunsaturated fatty acids, including arachidonic acid (C20:4) and docosahexaenoic acid (DHA, C22:6) (Fig. 1C). Quantification of individual phospholipid species confirmed the increases of most PC (2~3-fold) and PE (3~10-fold) species in the differentiated cells (Fig. 1D,E). These results suggest that the biosynthesis of phospholipids, particularly PE with polyunsaturated fatty acids, is elevated during osteoclast differentiation.

### Increase of cell surface PE during osteoclast differentiation

**Figure 2:**
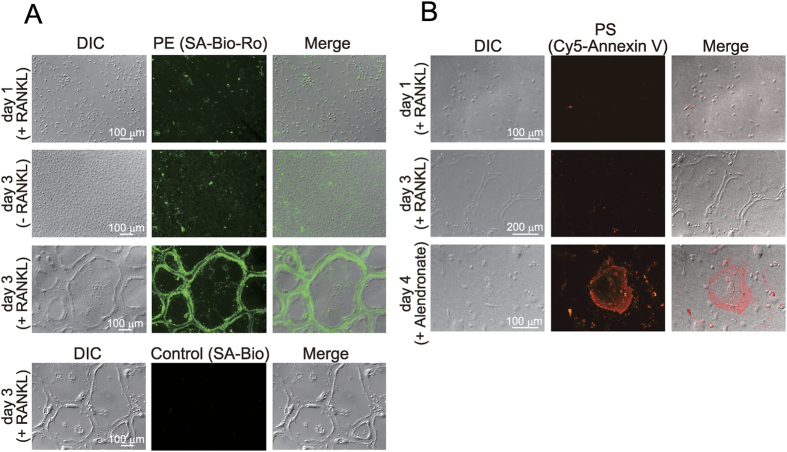
Increase of PE on the cell surface during osteoclast differentiation. Osteoclast precursors were cultured for 1 day or 3 days with M-CSF in the presence or absence of RANKL, and then exposure of PE (**A**) and PS (**B**) on the cell surface was assessed. (**A**) PE staining with SA-Bio-Ro. The cells were treated with cell-impermeable SA-Bio-Ro for 30 min and fixed. SA-Bio-Ro bound to the cell surface was stained with a FITC-conjugated anti-streptavidin antibody. DIC, difference interference contrast. For control, differentiated osteoclasts were treated with SA-Bio and immunostained. **(B)** PS staining with Cy5-conjugated annexin V. For positive control, differentiated osteoclasts (day 3) were cultured for an additional 1 day with 10 μM alendronate to induce apoptosis.

To address the distribution of PE during osteoclastogenesis, we visualised PE in the osteoclast membrane using Ro09–0198, a tetracyclic polypeptide that binds specifically to PE^18^, conjugated with streptavidin-biotin (SA-Bio-Ro). Since SA-Bio-Ro has no membrane permeability, it has been used as a convenient probe for detection of PE residing in the outer, but not inner, leaflet of the plasma membrane^14^. When osteoclast precursors were cultured with RANKL and M-CSF for 1 day, or with M-CSF in the absence of RANKL for 3 days, the fluorescence signal for PE on the cell surface was rather faint (Fig. 2A). In sharp contrast, mature osteoclasts induced by RANKL and M-SCF for 3 days exhibited a robust fluorescence signal in outer edges of the cells (Fig. 2A). No fluorescence staining was observed with SA-Bio, a control conjugate, confirming that the prominent FITC staining with SA-Bio-Ro in mature osteoclasts is specific for binding of Ro09–0198 to the cell surface (Fig. 2A). These results suggest that PE is exposed on the outer leaflet of the plasma membrane during osteoclast maturation.

In most mammalian cells, PS is localised preferentially in the inner leaflet of the plasma membrane and externalised to the outer leaflet during apoptosis^19^. The signal for annexin V, a PS-binding protein that detects externalised PS^20^, was barely evident in both undifferentiated and differentiated osteoclasts, even though prominent annexin V staining was obvious in osteoclasts undergoing apoptosis by treatment with alendronate^21^ (Fig. 2B). These results indicate that PE is specifically externalised on the plasma membrane of mature osteoclasts.

**Figure 3:**
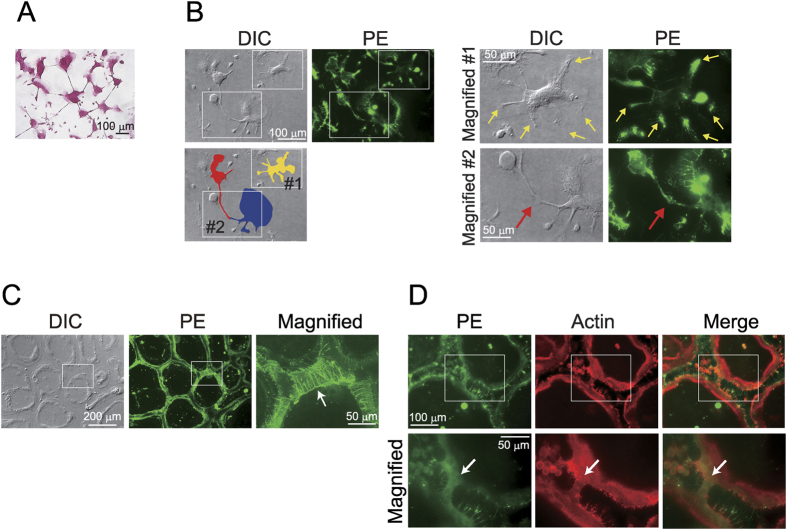
Localisation of PE on the surface of fusing filopodia during osteoclastogenesis. (**A**) TRAP staining of pre-osteoclasts. Osteoclast precursors were cultured for 2 days with M-CSF and RANKL, and then stained for TRAP. The cells protruded long filopodia and formed contacts with neighbouring precursor cells. (**B**) Distribution of PE in osteoclast precursors in the earlier stage of multinucleation. Osteoclast precursors undergoing cell-cell fusion were treated with SA-Bio-Ro and immunostained. Areas in white boxes in the left panels are magnified in the right panels. A cell coloured in yellow (#1) extended several filopodia where PE was localised to their surface (yellow arrows in Magnified #1). Red and blue coloured cells (#2) contacted with each other via long filopodia. PE staining was observed on the surface of the protrusions (red arrow in Magnified #2). **(C)** Multinucleated cells in the later stage extended a number of filopodia and formed contacts with their neighbouring cells (white arrow). (**D**) Distribution of PE and actin at foci of cell fusion. SA-Bio-Ro-treated osteoclasts were immunostained for streptavidin (green) and actin (red). Areas in white boxes in the top panels are magnified in the bottom panels. Arrows point to the region of cell fusion.

Prior to cell-cell fusion, osteoclast precursors protruded “neurite-like” filopodia and formed contacts with their neighbouring precursor cells (Fig. 3A). To obtain further details of PE localisation during osteoclastogenesis, we stained PE on the cell surface at the stages of osteoclast multinucleation. It was noteworthy that, in the earlier stage of multinucleation, PE was localised preferentially to the outer leaflet of filopodia in osteoclast precursors (Fig. 3B, Magnified #1, yellow arrows) and in cells contacted with each other via extended filopodia (Fig. 3B, Magnified #2, red arrows). Furthermore, multinucleated precursors in the later stage of osteoclast differentiation were in contact with neighbouring precursors via a number of filopodia, and PE was concentrated on the surface of the filopodia (Fig. 3C, arrow). During the cell-cell fusion process, fusing cells adhere to their partner cells and then actin accumulates at the site of fusion^22^. When two osteoclasts were in the process of fusion, both actin and PE signals were distributed at foci of cell fusion (Fig. 3D, arrows), suggesting that PE was exposed at the cell surface actin foci. Thus, PE on the cell surface is preferentially localised to filopodia where cell-cell fusion takes place.

### Cell surface PE is essential for osteoclast fusion

**Figure 4:**
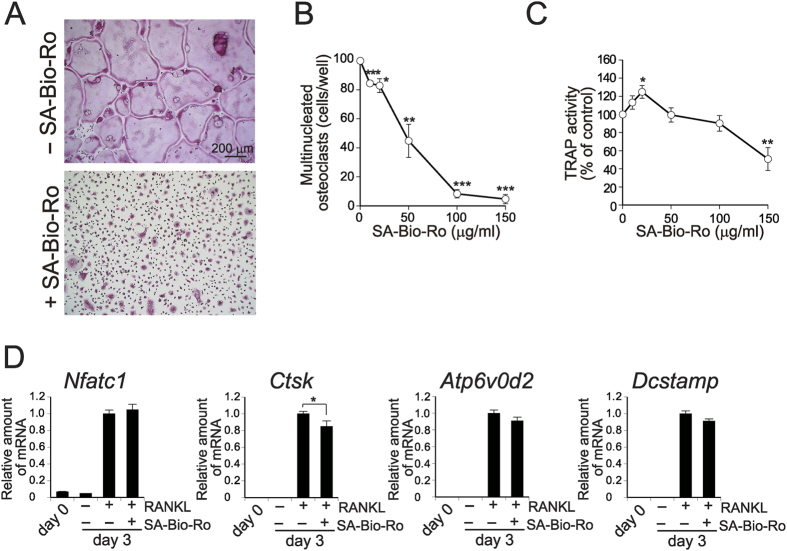
Blocking of osteoclast fusion by immobilisation of cell surface PE. Osteoclast precursors cultured for 2 days with RANKL and M-CSF were incubated with 100 μg/ml **(A)** or various concentrations (**B** and **C**) of SA-Bio-Ro for 1 h, washed, and cultured for additional 1 day. **(A)** TRAP staining of SA-Bio-Ro-treated cells. (**B**) The number of TRAP-positive multinucleated osteoclasts (n = 4). **(C)** TRAP activity of SA-Bio-Ro-treated cells (n = 7). (**D**) Gene expressions of osteoclast differentiation markers. Osteoclast precursors (day 0) were cultured with M-CSF in the presence or absence of RANKL for 3 days with or without SA-Bio-Ro treatment (100 μg/ml) on day 2. Total RNAs from the cells were reverse-transcribed and quantitative PCR for indicated genes was performed (n = 5 to 9). Each value is the mean ± SE.

To examine whether the exposure of PE on the cell surface is functionally involved in osteoclast fusion, mononucleated osteoclast precursors were treated with SA-Bio-Ro, which immobilises cell surface PE in culture cells^14^. Notably, SA-Bio-Ro inhibited multinucleation of osteoclasts with an IC_50_ of 45 μg/ml (Fig. 4A,B), whereas TRAP activity was insensitive at this concentration and blocked partially only at the highest concentration of 150 μg/ml (Fig. 4C). Expression levels of a panel of genes encoding osteoclast differentiation markers, including nuclear factor of activated T cells (NFAT) c1, cathepsin K, v-ATPase V_0_ domain and DC-STAMP (encoded by *Nfatc1, Ctsk, Atp6v0d2* and *Dcstamp*, respectively), were unaffected or decreased only modestly in cells treated with SA-Bio-Ro (Fig. 4D). These results suggest that the SA-Bio-Ro-treated cells still retained osteoclast signatures, but failed to become multinucleated. Thus, it appears that translocation of PE to the outer leaflet of the plasma membrane is essential for osteoclast fusion during osteoclastogenesis.

### Screening of lipid-related molecules whose expressions are correlated with PE dynamics

**Figure 5:**
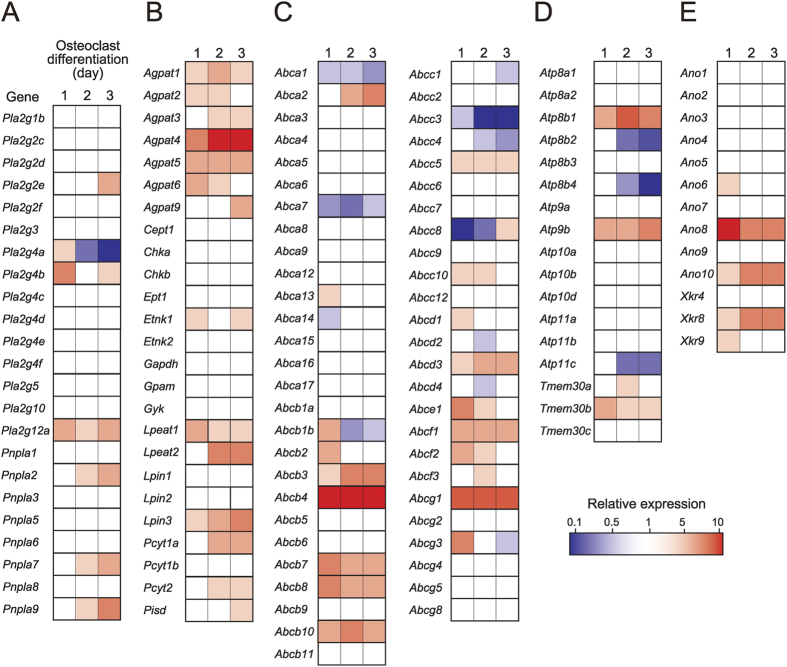
Gene expression profiles of lipid-related molecules during osteoclastogenesis. Osteoclast precursors (day 0, control) were cultured with M-CSF and RANKL for 1 to 3 days. Total RNAs were extracted at each time point and taken for quantitative PCR for individual genes. The oligonucleotide primers used are summarised in Supplementary Table 1. Relative mRNA levels of the lipid-related molecules are presented as heat maps. **(A)** lipid-degrading enzymes, (**B**) lipid-biosynthetic enzymes, (**C**) ABC transporters, (**D**) P-type ATPases, (**E**) anoctamins and Xkr proteins.

To determine the molecules responsible for PE dynamics during osteoclastogenesis, we screened 130 molecules related to lipid (particularly PE) biosynthesis, lipid degradation and transbilayer lipid transport using quantitative PCR. These included acyltransferases, phospholipid synthases, lipid phosphatases, phospholipases, ABC transporters, P-type ATPases and scramblases. Heat map visualisation (fold changes) of individual genes during osteoclast differentiation and their precise expression levels in osteoclasts are summarised in Fig. 5 and Supplementary Figs 1 and 2, respectively. Among the molecules screened, we focused on several genes whose expression levels were high in osteoclasts and altered markedly during osteoclastogenesis. These genes were 1-acylglycerol-3-phosphate *O*-acyltransferase 4 (AGPAT4; encoded by *Agpat4*), which transfers a fatty acyl residue to lysophosphatidic acid to produce phosphatidic acid, a major precursor for PE as well as PC, PS and PI^23^; acyl-CoA:lysophosphatidylethanolamine acyltransferase 2 (LPEAT2; encoded by *Lpeat2*), a member of the LPLAT family implicated in PE biosynthesis^24^; CTP:phosphoethanolamine cytidylyltransferase 2 (PCYT2; encoded by *Pcyt2*), which catalyses the production of CDP-ethanolamine, a precursor of PE in the *de novo* biosynthetic pathway^9^; PS decarboxylase (encoded by *Pisd*), which converts PS to PE; ABCA2, ABCB4 and ABCG1 (encoded by *Abca2, Abcb4* and *Abcg1*, respectively), members of the ABC transporters implicated in outward translocation of phospholipids^25^; ATP8B4 (encoded by *Atp8b4*), a putative membrane flippase^26^, and anoctamin 6 (Ano6, also known as TMEM16F; encoded by *Ano6*), a phospholipid scramblase^27^. All of these genes were expressed at high levels and prominently induced or downregulated during osteoclastogenesis (Fig. 5 and Supplementary Figs 1 and 2). To determine whether these lipid-related molecules are responsible for PE dynamics during osteoclastogenesis, we produced retroviruses that expressed shRNA-targeting mRNAs for these molecules, infected them into osteoclast precursors, and cultured the cells with RANKL and M-CSF for subsequent analysis.

### LPEAT2 is responsible for PE biosynthesis and osteoclast formation

**Figure 6:**
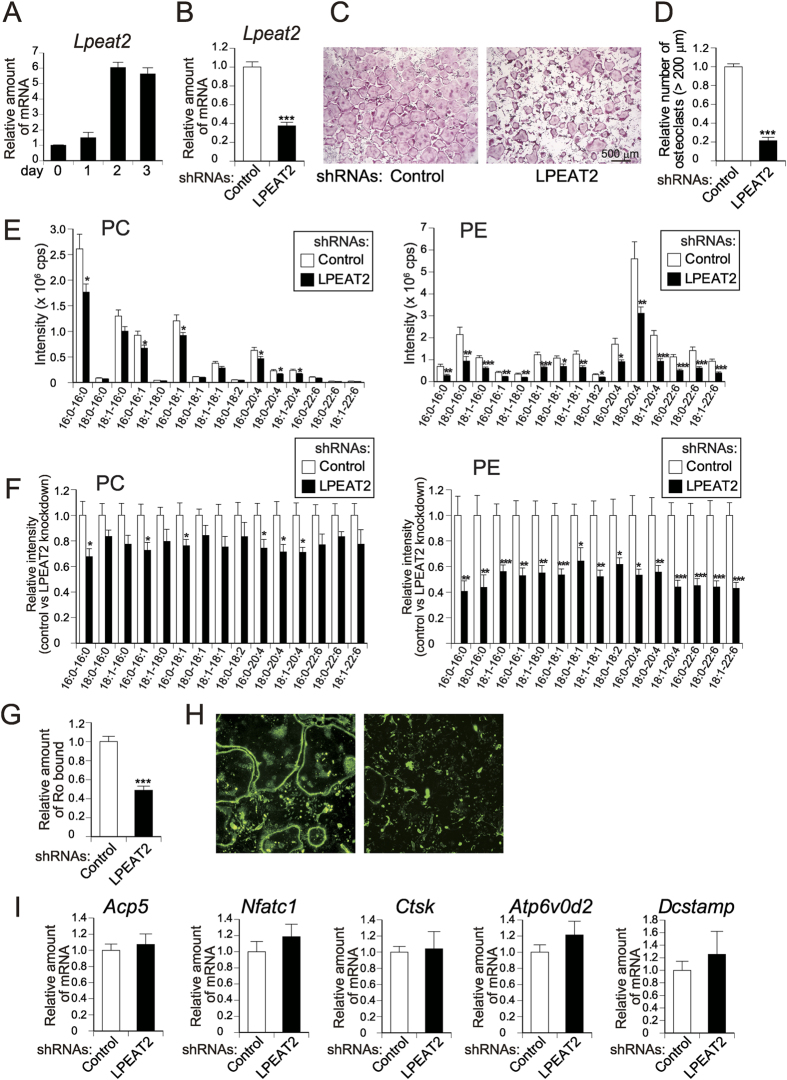
Role of LPEAT2 in PE biosynthesis and osteoclast formation. **(A**) Gene expression of *Lpeat2* during osteoclastogenesis. Osteoclast precursors (day 0) were cultured with M-CSF and RANKL for 1 to 3 days and subjected to quantitative PCR analysis (n = 3). (**B–I)** shRNA-mediated knockdown of LPEAT2 in osteoclasts. Osteoclast precursors were infected with retrovirus expressing shRNA for LPEAT2 or control and then cultured with M-CSF and RANKL for 5 days. (**B**) Efficiency of shRNA-mediated knockdown was validated by quantitative PCR (n = 8). The cells infected with the shRNA-bearing retroviruses were stained for TRAP (**C**) and TRAP-positive multinucleated osteoclasts were counted (n = 8) (**D**). (**E**) The shRNA retrovirus-infected cells were subjected to MS analysis. Individual PC and PE species are quantified (n = 8). (**F**) Relative amounts (control *versus* LPEAT2 knockdown, with control as 1) of the individual phospholipid species quantified in (**E**) (n = 8). (**G**) The cells infected with the shRNA-bearing retrovirus were treated with SA-Bio-Ro and immunostained, and then the fluorescence intensity was quantified (n = 8). Representative fluorescence images are shown in **(H)**. **(I)** Expressions of osteoclast-related genes in the shRNA retrovirus-infected cells (n = 12). Each value is the mean ± SE.

Among the lipid-biosynthetic or -degrading enzymes screened thus far, expression of *Lpeat2* was relatively higher than that of others and markedly induced during osteoclastogenesis (Fig. 6A and Supplementary Fig. 1). The kinetic increase of *Lpeat2* expression occurred in parallel with that of PE proportion (Supplementary Fig. 3A), which preceded induction of osteoclast differentiation markers (*Acp5* and *Dcstamp*) (Supplementary Fig. 3B), raising the possibility that LPEAT2 might be responsible for the increased PE production in the process of osteoclast differentiation. When *Lpeat2* expression was silenced by retrovirus-mediated transfer of its specific shRNA (to 37% of control) (Fig. 6B), formation of TRAP-positive multinucleated osteoclasts was greatly inhibited (to 21% of control), even though the cells were still stained for TRAP (Fig. 6C,D). Another LPEAT2-directed shRNA also inhibited osteoclast formation (Supplementary Fig. 3C,D), ruling out the possibility of an off-target effect. MS-based quantification of individual PC and PE species revealed that most PC species were reduced only modestly (to approximately 70–80% of control), while most PE species showed greater reduction (to approximately 40–60% of control) by LPEAT2 knockdown (Fig. 6E,F), suggesting that LPEAT2 synthesises PE in preference to PC with no apparent selectivity for fatty acid incorporation in differentiating osteoclasts. Moreover, the inhibition of PE synthesis by LPEAT2 knockdown was accompanied by marked reduction of PE exposure on the cell surface (to 49% of control) during osteoclast formation (Fig. 6G,H). However, knockdown of LPEAT2 did not affect the expressions of *Acp5* (encoding TRAP), *Nfatc1, Ctsk, Atp6v0d2* and *Dcstamp* (Fig. 6I), reminiscent of the results of SA-Bio-Ro treatment, which did not affect the expressions of these genes for osteoclast differentiation and function (Fig. 4D). In contrast, shRNA knockdown of PCYT2 and PS decarboxylase failed to block osteoclast formation or PE exposure on the cell surface (Supplementary Fig. 4), suggesting that these PE-biosynthetic enzymes are dispensable for the PE dynamics in osteoclasts. Although introduction of AGPAT4-targeted shRNA caused a slight inhibition of osteoclast formation, it did not affect the localisation of PE (Supplementary Fig. 4). Taken together, these results suggest that LPEAT2 is the major contributor to PE production that is linked to the increased PE exposure on the cell surface during osteoclastogenesis.

### ABCB4 and ABCG1 are responsible for PE relocalisation and osteoclast formation

**Figure 7:**
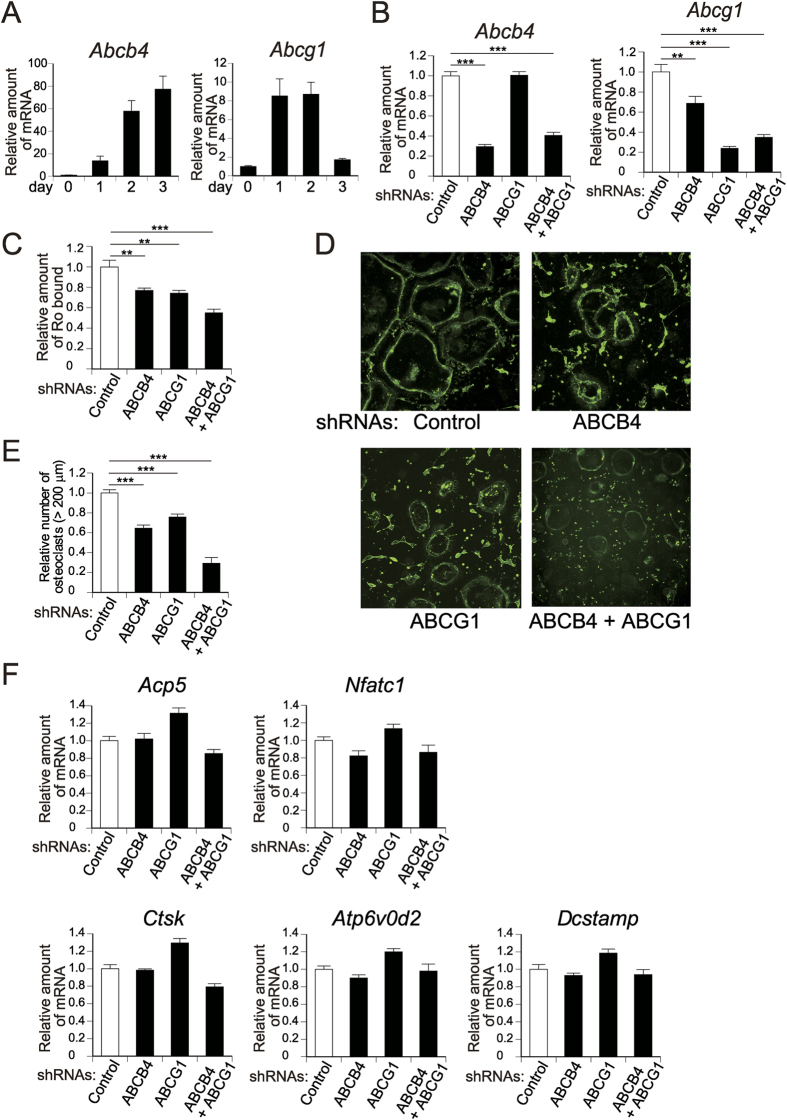
Role of ABCB4 and ABCG1 in PE relocalisation and osteoclast formation. (**A**) Gene expressions of *Abcb4* and *Abcg1* during osteoclastogenesis as analysed by quantitative PCR (n = 3 to 5). (**B–F**) shRNA-mediated knockdown of ABCB4 and ABCG1 in osteoclasts. Osteoclast precursors were infected with retroviruses expressing shRNAs for ABCB4 and ABCG1 (alone or in combination) or control and then cultured. (**B**) Efficiency of shRNA-mediated knockdown was validated by quantitative PCR (n = 10). (**C**) The shRNA retrovirus-infected cells were treated with SA-Bio-Ro, immunostained, and quantified the fluorescence intensity (n = 10 to 12). Representative fluorescence images are shown in (**D**). (**E**) The shRNA retrovirus-infected cells were stained for TRAP, and TRAP-positive multinucleated osteoclasts were counted (n = 12). (**F**) Expressions of osteoclast-related genes in the shRNA retrovirus-infected cells (n = 6). Each value is the mean ± SE.

To determine the molecule(s) responsible for PE translocation to the cell surface during osteoclastogenesis, we next performed shRNA knockdown analysis of lipid flip-flop proteins. Among these transporters, mRNA expressions of ABCB4 and ABCG1 were elevated (yet with different kinetics) during osteoclastogenesis (Fig. 7A), and their expression levels were relatively high compared to other ABC transporters (Supplementary Fig. 2). We noted that knockdown of either ABCB4 or ABCG1 resulted in marked reduction (to ~30% of control) of its expression (Fig. 7B), which was accompanied by modest but significant reduction of PE exposure on the cell surface (to 77% and 74% of control, respectively) (Fig. 7C). When both ABCB4 and ABCG1 were simultaneously silenced, the suppression of PE relocalisation was even more prominent (to 55% of control) (Fig. 7B,C). Importantly, these partial impairments of PE exposure on the cell surface in the knockdown cells resulted in greater reduction of the number of multinucleated osteoclasts formed (to 65%, 76% and 29% of control by silencing of ABCB4, ABCG1 and both, respectively) (Fig. 7D,E). Experiments using the second ABCB4- or ABCG1-directed shRNA also gave similar results (Supplementary Fig. 5A,B). Neither LPEAT2 expression nor PE abundance and composition was profoundly affected by double knockdown of ABCB4 and ABCG1(Supplementary Fig. 5C,D), further supporting that LPEAT2 and ABCB4/ABCG1 act on distinct steps of PE dynamics, *i.e*. its biosynthesis and transport, respectively. On the other hand, the expressions of *Acp5, Nfatc1, Ctsk, Atp6v0d2* and *Dcstamp* were unaffected or changed only slightly by knockdown of these ABC transporters (Fig. 7F), suggesting that the reduction of ABCB4 or ABCG1 impairs only the cell-cell fusion process but does not affect cell differentiation *per se* during osteoclast formation. In contrast, shRNA knockdown of other lipid transporters, including ABCA2 (another potential floppase), ATP8B4 (a potential flippase whose downregulation could be linked to PE exposure) and Ano6 (a scramblase whose upregulation could be linked to PE exposure), affected neither osteoclast formation nor PE relocalisation on the cell surface (Supplementary Fig. 4), thus ruling out the possibility that they contributed to PE translocation. In conclusion, these results suggest that ABCB4 and ABCG1 are responsible for the translocation of PE to the external surface, which has profound influence on osteoclast formation, most likely cell-cell fusion, at the final stage of osteoclastogenesis.

## Discussion

In this study, we have provided evidence that alteration in the asymmetric distribution of PE in the plasma membrane is crucial for proper osteoclast homeostasis. During *ex vivo* osteoclastogenesis, PE, which otherwise resides in the inner leaflet of the plasma membrane, is highly exposed on the cell surface, particularly in filopodia, of osteoclasts undergoing cell-cell fusion. shRNA-based knockdown studies have revealed that the PE-biosynthetic enzyme LPEAT2 and the phospholipid floppases ABCB4 and ABCG1, which are markedly induced during osteoclastogenesis, lead to increased biosynthesis and mobilisation of PE and thereby promotion of cell-cell fusion.

Biological membranes exhibit various function-related shapes, and membrane curvature defines the morphology of the plasma membrane and intracellular organelles^13^. Phospholipids have intrinsic head groups, which are critical determinants of membrane curvature depending on their structures. Both PC and SM have a larger choline head group and tend to form convex structures when the lipids pack together. In contrast, PE has a smaller head group and packs into concave structures^13^. Therefore, in the plasma membrane of typical spheroidal cells, PC and SM tend to be localised in the outer leaflet to form a convex shape, whereas PE localises preferentially to the inner leaflet with a concave shape. Although PS is cylindrical and has no preferred curvature, this negatively charged phospholipid is present predominantly in the inner leaflet of the plasma membrane and contributes to electrostatic association with cationic proteins beneath the membrane^28^. Because the asymmetrical distribution of phospholipids stabilises cell morphology, disruption of the asymmetrical topology changes the membrane curvature, thus altering the cell shape. For instance, PS is exposed on the surface of erythrocytes in humans with sickle cell disease^29^ and in mice deficient in ATP11C, a flippase, which results in erythrocytes having an abnormal shape^30^. In addition, PE is enriched in the cell surface of the cleavage furrow during cytokinesis^14^. Since filopodia are generally linked to cell motility and cell-cell interaction^31^, they undergo continuous alterations in morphology so as to elongate, retract, and turn in direction. Hence, it is reasonable to assume that membrane curvature of filopodia changes dynamically to ensure their flexibility.

We have shown in this study that osteoclast precursors elongate their filopodia, which form contacts with neighbouring precursors. In accordance with this, PE loses its asymmetric distribution, becoming exposed on the outer leaflet of filopodia. Furthermore, immobilisation of PE in mononucleated precursors inhibits cell-cell fusion, demonstrating that the mobility of PE in filopodia is pivotal for osteoclast fusion. Biophysical studies have shown that PE in the outer leaflet of the plasma membrane favours invagination for membrane budding because PE prefers to pack into concave structures^32^, that PE increases the formation of lipid buds and tubules in synthetic lipid vesicles^33^, and that PE promotes formation of the hemifusion intermediate during cell-cell fusion^15^. Therefore, disruption of the asymmetric distribution of PE in filopodia favours filopodium formation and cell-cell fusion. Accordingly, we conclude that the PE dynamics during osteoclastogenesis are essential for appropriate filopodium formation, followed by osteoclast fusion.

Although no unifying mechanisms underlying the various cell-cell fusion events have yet been proposed, it is likely that cell fusion is accompanied by redistribution of PE in the plasma membrane. Indeed, in fusing chick embryonic myoblasts, PE is concentrated in the outer leaflet of the plasma membrane^34^. Besides PE, PS is also exposed on the cell surface of myoblasts^34^, although the functional importance of PS exposure in myoblasts has been obscure. Since PS is not exposed on the cell surface during osteoclastogenesis in the present study, the contribution of PS to osteoclast fusion seems unlikely. Biophysically, the head group of PE is less hydrated, so that adjacent PE-rich lipid bilayers can easily interact with each other, whereas PS-rich bilayers repel each other because of their electrostatic and hydration properties^35^. PE facilitates membrane fusion much more efficiently than PC and PS in liposome-liposome and virus-liposome fusion models^36^,^37^. Given that PS exposed on apoptotic cells is recognised by macrophages as an “eat-me” signal^27^, it is conceivable that PE and PS on the cell surface may have general and distinct roles as mediators of cell fusion and apoptotic phagocytosis, respectively.

We demonstrate that the PE dynamics during osteoclastogenesis consist of at least two steps; increase in PE synthesis by LPEAT2 and translocation of PE by ABCB4 and ABCG1. LPEAT2 is a member of the LPLAT family in the Lands’ cycle pathway^23^, and has been reported to produce PE in preference to other phospholipids *in vitro*^24^, although its physiological function is still largely unknown. LPEAT2-knockdown experiments revealed that this enzyme indeed preferentially generates PE to PC during osteoclastogenesis. To our knowledge, our study is the first to identify the role of LPEAT2 in PE biosynthesis in a physiologically relevant context *ex vivo*, and will be a foundation to find the physiological function(s) of this enzyme *in vivo* in the future study. It is unlikely that LPEAT1, another PE-biosynthetic enzyme in the Lands’ cycle^38^, is a major contributor to PE synthesis for osteoclast fusion, because its expression level is low and shows reciprocal correlation with osteoclastogenesis. Furthermore, none of the enzymes in other PE-biosynthetic pathways tested, including those in the PS decarboxylation and the CDP-ethanolamine pathways^9^, affects osteoclast fusion and differentiation. Taken together, these results suggest that LPEAT2 plays a principal role in PE production during osteoclastogenesis and that the enrichment of PE with polyunsaturated fatty acids (arachidonic acid and DHA) may contribute to increased membrane fluidity, leading to acceleration of filopodium formation.

In addition to the increased PE biosynthesis by LPEAT2, here we demonstrate that ABCB4 and ABCG1 are responsible for the outward translocation of PE during osteoclastogenesis. The ABC transporter superfamily consists of about 50 molecules, among which at least 10 members are known to translocate phospholipids from the inner to the outer leaflet of the plasma membrane^39^. ABCB4 transports PC, PE, SM and cholesterol *in vitro*^25^ and is crucial for the efflux of cholesterol and phospholipids into bile *in vivo*^39^. The expression level of *Abcb4* is increased approximately 80-fold in differentiating osteoclasts, suggesting its functional linkage to osteoclastogenesis. Indeed, a partial reduction of PE exposure by shRNA-mediated knockdown of ABCB4 leads to more profound inhibition of osteoclast fusion. It has been reported that ABCB4-deficient mice exhibit reductions of both bone mineral content and bone density, which are ascribed to impairment of intestinal vitamin D absorption due to reduction of hepatic bile secretion^40^. The paucity of vitamin D in these mice is so severe that the loss of bone formation caused by vitamin D insufficiency may overcome the decrease in bone absorption resulting from impairment of osteoclast formation associated with ABCB4 deficiency. ABCG1, in cooperation with ABCA1, effluxes cholesterol and PC into high-density lipoproteins and also regulates lipid homeostasis in various cells^25^. Although ABCG1 can efflux PC, PS, SM, cholesterol and vitamin E^25^,^41^, it remains controversial whether this transporter mediates efflux of PE^42^. Our present findings may represent the first evidence that ABCG1 transports PE to facilitate cell fusion in cooperation with ABCB4. It should be noted that inhibition of cholesterol efflux from late endosomes blocks osteoclast formation^43^, raising the possibility that the suppression of osteoclastogenesis by ABCB4 or ABCG1 knockdown is caused by the reduction of cholesterol efflux rather than or in addition to the impairment of PE exposure. Nevertheless, our results that osteoclast fusion is inhibited by immobilisation of cell surface PE with SA-Bio-Ro treatment, which does not affect cholesterol distribution, and that PE exposure is also reduced by LPEAT2 knockdown, resulting in inhibition of osteoclast formation, imply the importance of cell surface PE for osteoclast fusion.

In conclusion, our results highlight that rearrangement of PE in the plasma membrane is essential for osteoclast fusion. An increase in PE synthesis by LPEAT2 and exposure of PE on the outer leaflet by the phospholipid floppases ABCB4 and ABCG1 are required for adequate filopodium formation and function, and thereby cell-cell fusion, in osteoclasts. Although *in vivo* studies to expand our findings will be needed, our present results nonetheless provide new insight into osteoclast biology.

## Methods

### Cell culture

C57BL/6 mice were purchased from Charles River Laboratories Japan (Yokohama). Bone marrow cells were isolated from C57BL/6 mice (4–7 weeks old, male) and cultured in α-minimum essential medium (α-MEM) containing 10% (v/v) FBS, 100 ng/ml M-CSF (Kyowa Hakko Kogyo) and 1 ng/ml TGF-β (R & D Systems) in 100-mm culture dishes. After 3 days of culture, floating cells were removed and attached cells were used as osteoclast precursors. To generate osteoclasts, the osteoclast precursors were cultured for 3 days in α-MEM containing 10% FBS, 150 ng/ml soluble RANKL (Oriental Yeast) and 50 ng/ml M-CSF.

### TRAP staining

Osteoclast differentiation was assessed by TRAP staining as described previously^44^. Briefly, cultured cells were fixed with 10% (v/v) formalin in PBS followed by ethanol/acetone (1/1, v/v), and stained for TRAP with 0.1 mg/ml naphthol AS-MX phosphate (Sigma) and 0.6 mg/ml fast red violet LB salt (Sigma) in 0.1 M sodium acetate buffer, pH 5.0, containing 50 mM sodium tartrate. The TRAP-positive cells were photographed with a BZ-8000 digital microscope (Keyence), and counted manually. In some experiments, TRAP activity was quantified as follows. Cells in 96-well plates were fixed and incubated for 5 min in 0.1 ml of 10 mM *p*-nitrophenyl phosphate (Sigma) in 0.1 M sodium acetate buffer, pH 5.0, containing 50 mM sodium tartrate. After incubation, the solution was transferred to a tube containing 0.1 ml of 0.1 N NaOH, and the absorbance at 405 nm was determined.

### Phospholipid composition analysis

Phospholipids were extracted from cells by the method of Bligh and Dyer^45^ and separated by two-dimensional thin layer chromatography on silica plates. The solvent systems used for chromatography were chloroform/methanol/acetic acid, 65:25:10 (v/v/v) in the first dimension and chloroform/methanol/formic acid, 65:25:10 (v/v/v) in the second dimension. Lipids were visualised with iodine vapour and identified by comparing their positions with those of authentic standard phospholipids. The individual spots were scraped off and the lipid phosphorus was quantified as described by Gerlach and Deuticke^46^.

### MS analysis

MS analysis was performed using a 4000Q-TRAP quadrupole-linear ion trap hybrid mass spectrometer (AB Sciex) with a NexeraX2 liquid chromatography system (Shimazu). The samples were separated by a step gradient with mobile phase A (acetonitrile/methanol/water, 1:1:1 (v/v/v), containing 5 μM phosphoric acid and 1 mM ammonium formate) and mobile phase B (2-propanol containing 5 μM phosphoric acid and 1 mM ammonium formate) at a flow rate of 80 μl/min at 50 °C. The detection of each lipid was performed by electrospray mass spectrometry and multiple reaction monitoring. PC and PE with C14:0-C14:0 (Avanti Polar Lipids) were used as internal standards for quantification.

### Fluorescence microscopy

Streptavidin (Jackson) was conjugated with biotinylated Ro09–0198 (provided by Dr. M. Umeda, Kyoto Univ., Japan) and separated from free biotinylated Ro09–0198 using a Sephadex G-25 gel filtration column (GE Healthcare). For staining of cell surface PE, cells were incubated with 50 μg/ml SA-Bio-Ro for 30 min in α-MEM containing 0.2% (w/v) fatty acid-free bovine serum albumin (BSA) (Sigma). The cells were washed with 20 mM HEPES, pH 7.4, 115 mM NaCl, 5.4 mM KCl, 2.2 mM CaCl_2_, 0.8 mM MgCl_2_ and 13.8 mM glucose, and then fixed with 4% (w/v) paraformaldehyde in PBS for 30 min, followed by a 5-min permeabilisation with 0.1% (v/v) Triton X-100 in PBS. Immunostaining was performed with a FITC-conjugated anti-streptavidin antibody (Vector Laboratories). F-actin was stained using Alexa Fluor 594-conjugated phalloidin (Molecular Probes). PS on the cell surface was detected with an annexin V-Cy5 apoptosis detection kit (BioVision) in accordance with the manufacturer’s protocol. Fluorescence microscopy was performed using a DSU-IX81 microscope (Olympus). The images were photographed with a C10600 digital camera (Hamamatsu Photonics). In some experiments, immunofluorescence intensity of cells cultured in 96-well plates was quantified with an ImageXpress Micro XL HCS (Molecular Devices) and MetaXpress Image Analysis software (Molecular Devices).

### Cell fusion arrest by SA-Bio-Ro

Osteoclast precursors were cultured for 2 days in α-MEM containing 10% FBS, 150 ng/ml soluble RANKL and 50 ng/ml M-CSF. The cells were then incubated with various concentrations of SA-Bio-Ro for 1 h in α-MEM containing 0.2% fatty acid free BSA, and washed with α-MEM containing 10% dialysed FBS. The cells were cultured for a further day in α-MEM containing 10% dialysed FBS and stained for TRAP as described above.

### Retrovirus production and infection

To construct shRNA-expressing vectors, shRNA oligonucleotide targeting sequences were inserted into a pSIREN-RetroQ-DsRed expressing vector (Clontech). The targeting sequences used were as follows: ABCA2, 5’-GCTGGATGCCCAGAAACTTCT-3’; ABCB4, 5’-GAGCTAGCTGCATATGCAA-3’; ABCG1, 5’-GGATCTCTGGGAAATTCAACA-3’; AGPAT4, 5’-GGGTAAGATGACTAAATTA-3’; Ano6, 5’-GCGAGAAGATTGGAATCTACT-3’; ATP8B4, 5’-CTCGAACTCCAGAGACAATTA-3’; LPEAT2, 5’-CGCGTGGCTCCAAGTAGCA-3’; PCYT2, 5’-GCTATGACATGGTGCATTATG-3’; PS decarboxylase, 5’-GTACAGGGAACGGAAGCTT-3’. A negative control shRNA-expressing vector was constructed in accordance with the manufacturer’s protocol (Clontech). Recombinant retroviruses were produced by co-transfecting the pSIREN-RetroQ-DsRed vectors together with an envelope expression vector, pVSV-G (Clontech), into GP2–293 pantropic packaging cells (Clontech) using Lipofectamine LTX (Invitrogen). The culture medium was replaced with fresh α-MEM containing 10% FBS for 8 h following transfection. The viral supernatants were harvested at 2 days after transfection and filtered with a 0.45-μm filter. For infection, osteoclast precursors (1.4 × 10^4^ cells) were plated on a 96-well plate. On the following day, the medium was replaced with 250 μl of the viral supernatant containing 5 μg/ml polybrene (Nacalai Tesque) and 100 ng/ml M-CSF. After 8 h, the viral supernatant was removed, and the cells were cultured in growth medium containing RANKL and M-CSF for subsequent analysis.

### Quantitative PCR analysis

Total RNA was extracted from cells or tissues using TRIsol reagent (Invitrogen), and reverse-transcribed using PrimeScript RT Master Mix (Takara Bio) in accordance with the manufacturer’s protocol. Quantitative PCR analysis was performed with a LightCycler 480 (Roche) using SYBR Premix Ex Taq II (Takara Bio). Oligonucleotide primers used in quantitative PCR analysis were designed using ProbeFinder software (Roche). In some experiments, quantitative PCR analysis was performed with a TaqMan Gene expression system and TaqMan probes (Applied Bioscience). The primers and probes used are listed in Supplementary Table 1.

### Statistics

Data were expressed as the mean ± SE. Statistical significance was assessed using unpaired Student’s *t* test (in the figures, statistical significance is denoted as follows: *p < 0.05, **p < 0.01, and ***p < 0.001).

### Study approval

Procedures carried out using mice were approved by the Institutional Animal Care Committee of Tokyo Metropolitan Institute of Medical Science, in accordance with the Standards Relating to the Care and Management of Experimental Animals in Japan.

## Additional Information

**How to cite this article:** Irie, A. *et al*. Phosphatidylethanolamine dynamics are required for osteoclast fusion. *Sci. Rep.* **7**, 46715; doi: 10.1038/srep46715 (2017).

**Publisher's note:** Springer Nature remains neutral with regard to jurisdictional claims in published maps and institutional affiliations.

## Supplementary Material

Supplementary File
