# Supplementary material for: Phosphatidylethanolamine dynamics are required for osteoclast fusion
Source: Sci Rep. 2017 Apr 24;7:46715. doi: 10.1038/srep46715 (PMC5402267; doi:10.1038/srep46715)
Supplement: Supplementary File [file srep46715-s1.pdf]

Supplementary information

Phosphatidylethanolamine dynamics are required for osteoclast fusion

Atsushi Irie[1], Kei Yamamoto[1, 2, 3], Yoshimi Miki[1] and Makoto Murakami[1, 4*]

[1]Lipid Metabolism Project, Tokyo Metropolitan Institute of Medical Science, Tokyo, Japan., [2]Faculity of Bioscience and Bioindustry, Tokushima University, Tokushima, Japan., [3]PRIME and [4]AMED-CREST, Japan Agency for Medical Research and Development, Tokyo, Japan.

*Address correspondence to: Makoto Murakami, PhD, Lipid Metabolism Project, Tokyo Metropolitan Institute of Medical Science, Setagaya-ku, Tokyo 156-8506, Japan. Tel: 81-3-5316-3228; Fax: 81-3-5316-3125; E-mail: murakami-mk@igakuken.or.jp

Supplementary Table 1. Oligonucleotide primers for quantitative PCR.

Gene	Sense primer	Antisense primer
Abca1	5'-gcagatcaagcatcccaact-3'	5'-ccagagaatgtttcattgtcca-3'
Abca2	5'-cgcaaagagacggacaagat-3'	5'-cagtgagtgccgcttgttag-3'
Abca3	5'-ctgcttggaaacaactggtg-3'	5'-ggagcaggaacgctgagat-3'
Abca4	5'-ccaccacctactggctgac-3'	5'-ccgcactcacagcataattc-3'
Abca5	5'-gcagaaattgaccaagcaga-3'	5'-aaggattttgaatctgtttcttcct-3'
Abca6	5'-gagcagcgtcagcgattat-3'	5'-cgaagggtagaggccagaa-3'
Abca7	5'-tcaaccatcctttgaacttgac-3'	5'-catccacagaggaggctatca-3'
Abca8	5'-cctggttccacctgctacat-3'	5'-atttcagaaaattcacgattctca-3'
Abca9	5'-gatccctaaaacaaaagagcaca-3'	5'-cacaaatcattttattgttcagtgg-3'
Abca12	5'-cctgctaaaccagacgatcc-3'	5'-acttgcacaaaggggttcc-3'
Abca13	5'-ccccagcacatcaagaaca-3'	5'-ttccttgtggagccaaactt-3'
Abca14	5'-ggggatgtgttcattgaagg-3'	5'-ggacagtagccaacttttgacc-3'
Abca15	5'-ctgctctggtatttgatctcattatc-3'	5'-aaatttgcagtatttgaacataacca-3'
Abca16	5'-cgacccaggaatccaaattat-3'	5'-ctgccaggaatccttcctta-3'
Abca17	5'-gccccgggaattttcata-3'	5'-ccaggaagccttctttgttgta-3'
Abcb1a	5'-gggcatttacttcaaacttgtca-3'	5'-tttacaagcttcatttcctaattcaa-3'
Abcb1b	5'-agtggacccaacagtactctgat-3'	5'-gcaccaatcccggtgtaata-3'
Abcb2	5'-aagaaggaggccttggctta-3'	5'-gcagcattcccgagacac-3'
Abcb3	5'-ggtggcctgctctccttc-3'	5'-ccgtacatgtaaaccaggttcc-3'
Abcb4	5'-cgactttgaactaggcagca-3'	5'-aacaggccaattaaattcactttc-3'
Abcb5	5'-tgtttctttcagctatccatcaag-3'	5'-tcttcagattgagacctttcagaac-3'
Abcb6	5'-cacgttcatgaagaagaccaag-3'	5'-gtggatcgtcggtctgttg-3'
Abcb7	5'-gatgatgcttgtcagtagtgttttg-3'	5'-gtgttcctagggttaccaatgc-3'
Abcb8	5'-tccaacatcgctttcaactg-3'	5'-tgaggtctcccccctttcag-3'
Abcb9	5'-atgccgagagcgaatacct-3'	5'-accgtgtgtctctgcaggtt-3'
Abcb10	5'-gatccaagattgggacagtga-3'	5'-gcaatgttctcagcgacaga-3'
Abcb11	5'-gctgcttttggtggtgaga-3'	5'-cagcgctgagcaaacataag-3'
Abcc1	5'-gccccagtgttactggtca-3'	5'-caaaaaggtggcgagcag-3'
Abcc2	5'-caaatccaattctctacctatgcac-3'	5'-gcctgcagtgttggatca-3'
Abcc3	5'-gctgagggtggggataatct-3'	5'-ggctcgggctaggcatac-3'
Abcc4	5'-gagcacacggacgaggag-3'	5'-tcttcaatggcctctttaagttg-3'
Abcc5	5'-gttctgggctctgacaggat-3'	5'-gaccgatggggtgtcaaa-3'
Abcc6	5'-catcttgccaggaatcaaca-3'	5'-gggctcctgacggaagtt-3'
Abcc7	5'-cagcagctcaaacaactgga-3'	5'-tgtcacaaggtgggtgaaaa-3'
Abcc8	5'-gggagatgcagagggtctc-3'	5'-ttctccctcgctgtctgg-3'
Abcc9	5'-tgcctttcgagtcctgtaaga-3'	5'-tcgtagctgtccaggtggta-3'
Abcc10	5'-cgccctcaatgatgatctaagt-3'	5'-ttctctccaacctctgtctgg-3'
Abcc12	5'-tccaaaggcttgtcattgtct-3'	5'-tgttcgcacacacacttgaa-3'
Abcd1	5'-gccagcctcaacatcagg-3'	5'-actcttgccacagccattg-3'
Abcd2	5'-gcgcggatgttttaccataa-3'	5'-gatgctcacggcactggta-3'
Abcd3	5'-gggagaagcagacaatccac-3'	5'-ccgaaagaaaatgaaattatgtagg-3'
Abcd4	5'-tgagtgggacttggacaaaa-3'	5'-cgatcgagcagaaaagctg-3'
Abce1	5'-gctgctttggctactgcac-3'	5'-tgtctgccataattgtaacaaagaat-3'
Abcf1	5'-ggacaagaagaccaaaaagacg-3'	5'-ccttctccttctgcaatttttc-3'
Abcf2	5'-ggacctcgatgcttgtgtg-3'	5'-accaggatgcgcttgaaa-3'

Abcf3	5'-ggagctcagcctcaggatt-3'	5'-ctcctccagcttcccataga-3'
Abcg1	5'-gggtctgaactgccctacct-3'	5'-tactcccctgatgccacttc-3'
Abcg2	5'-gccttggagtactttgcatca-3'	5'-aaatccgcagggttgttgta-3'
Abcg3	5'-agcattcctcattatggatttagg-3'	5'-tgtgttgtgttctggacaaaagt-3'
Abcg4	5'-tgatgtgcccttccaggt-3'	5'-caaggctgagaagagcagga-3'
Abcg5	5'-tcctgcatgtgtcctacagc-3'	5'-atttgcctgtcccacttctg-3'
Abcg8	5'-aaccctgcggacttctacg-3'	5'-ctgcaagagactgtgccttct-3'
Acp5	5'-cgtctctgcacagattgcat-3'	5'-aagcgcaaacggtagtaagg-3'
Actb	5'-gaatctgcatgggcaacc-3'	5'-accagaggcatacagggaca-3'
Agpat1	5'-aaaggaaagacgcttcacctc-3'	5'-gtgtcagcccttctgtgga-3'
Agpat2	5'-gtgctctgcctgtccttctc-3'	5'-acgaaccagctgatgatgc-3'
Agpat3	5'-ggaggaaaacacctgtccac-3'	5'-tcaagggtgtccgacctg-3'
Agpat4	5'-cgcaagatcaatgccagac-3'	5'-accactccagaagcatcacc-3'
Agpat5	5'-ctagcgaatcatcaaagcaca-3'	5'-tctttcagtacgtagcgcaca-3'
Agpat6	5'-gagtgctgattcggtattgct-3'	5'-cactaccaagaggccaatcc-3'
Agpat9	5'-gtgctgggtgtcctagtgc-3'	5'-aagctgatcccaatgaaagc-3'
Ano1	5'-accaaggccaagtacagcat-3'	5'-tgcagctgagtatacgccatt-3'
Ano2	5'-cagccacataagcctatccag-3'	5'-cagggagcagagggatgtc-3'
Ano3	5'-ctggggaatgtggtacaagc-3'	5'-tcttctcaccaaagtacatcctga-3'
Ano4	5'-tggcttcatttttgctgttct-3'	5'-cctgcttatttgtttatcgatcc-3'
Ano5	5'-tcctgaggaggcgtcttatg-3'	5'-ccatctctgaagaagaccgagt-3'
Ano6	5'-tcactggagaatcagcaggac-3'	5'-gggcttcccgttaaattctt-3'
Ano7	5'-ttggaatccgaaatgaggag-3'	5'-gagctcctgtgccagctc-3'
Ano8	5'-cttggaggaccagccaatc-3'	5'-tgaactggaaacacctgctg-3'
Ano9	5'-tctcacacctgccagtgc-3'	5'-gatctgggaactctcatcatcc-3'
Ano10	5'-ctgattgtggtggccgtag-3'	5'-tggcaaatgcgagtatgaac-3'
Atp6v0d2	5'-aagcctttgtttgacgctgt-3'	5'-gccagcacattcatctgtacc-3'
Atp8a1	5'-agaaatggtgcatgggaaat-3'	5'-ccttcactatctcccccactg-3'
Atp8a2	5'-cttccagcagacatggtcct-3'	5'-gcagtttcaacatagcacatcc-3'
Atp8b1	5'-agtttgcctgtgctcctcat-3'	5'-gaggctcagcttgtcactca-3'
Atp8b2	5'-caacaaatgctccatcaacg-3'	5'-ctttgtgccccaagacatc-3'
Atp8b3	5'-gtcttgaaacagcgccctaa-3'	5'-ggctccacggctacttctt-3'
Atp8b4	5'-ttgtggaaagtgagggaagg-3'	5'-cctgagttttcaccacgtca-3'
Atp9a	5'-gaagcgggtggacagtagg-3'	5'-tgtcccaaccagacagtacg-3'
Atp9b	5'-atgagaaccgcacctaccag-3'	5'-tctgtccatcgtaccagtgc-3'
Atp10a	5'-cgcctggagacgaacttg-3'	5'-ttcctgaaggcggtcttctat-3'
Atp10b	5'-accactgagcccagaaagag-3'	5'-tccaaggatgtccctagagc-3'
Atp10d	5'-gaggtggtgaaattggttcg-3'	5'-atcattggcaccgtcacc-3'
Atp11a	5'-ggcaacggagagaactcaga-3'	5'-agaggccagaggggtgaa-3'
Atp11b	5'-tcctacaagtacagaaaaggcaca-3'	5'-acagcacacggagtccaag-3'
Atp11c	5'-agttgtaaagaatgttcgaagaagaa-3'	5'-tcagatgcccttctacagctc-3'
Cept1	5'-ggaacattgcgatttggaata-3'	5'-gttcctgagagaagctggaataa-3'
Chka	5'-caactgcacaagatcctctcttac-3'	5'-gatctagtatactgcagcaatgacct-3'
Chkb	5'-cactatagagttcggctacttgga-3'	5'-gatgatggggaactcgtcag-3'
Ctsk	5'-cgaaaagagcctagcgaaca-3'	5'-tgggtagcagcagaaacttg-3'
Dcstamp	5'-cgaagctccttgagaaacga-3'	5'-ggactggaaaccagaaatgaa-3'
Ept1	5'-cctgacatacttcgaccctga-3'	5'-caaacccagtcaggcacat-3'
Etnk1	5'-ttgctgatgaaaacattaataaaagg-3'	5'-aagcagctctttcatccaagtc-3'

Etnk2	5'-ttatcttgaggcgcagaagg-3'	5'-gcccagaagaaatgagatgc-3'
Gapdh	5'-ttccaggaaataataactttgtcaag-3'	5'-cactgcactgaaatacgtgct-3'
Gpam	5'-ggaaggtgctgctattcctg-3'	5'-tgggatactggggttgaaaa-3'
Gyk	5'-ggcaaagagcgagtgagc-3'	5'-ggctctcacgtcaaattcct-3'
Lpeat1	5'-tcctaactggagtccctgtca-3'	5'-tggaagagaggaagtggtgtc-3'
Lpeat2	5'-ccgttcgtgcatgagttaca-3'	5'-attggggccagcagtacc-3'
Lpin1	5'-cccttctatgctgcttttgg-3'	5'-gggacactcccacttgctt-3'
Lpin2	5'-tgaggtccctccagcaaat-3'	5'-gaagtgtcattctctgcaggtct-3'
Lpin3	5'-gactactccagagagccatactcc-3'	5'-ggcccaagtgttggtttct-3'
Nfatc1	5'-tccaaagtcattttcgtgga-3'	5'-ctttgcttccatctcccaga-3'
Pcyt1a	5'-ggtccagaaggagcactgaa-3'	5'-ctctgcttgggactgatgg-3'
Pcyt1b	5'-tctccagagagggtacacagc-3'	5'-tggaaacggtacttcttctcg-3'
Pcyt2	5'-aagggtccgggagatcag-3'	5'-tggagtggccataatgcac-3'
Pisd	5'-ttgcctcttcaggcaggt-3'	5'-cgcagcaggaacaaggat-3'
Pla2g2d	5'-gctctgggctggaactatga-3'	5'-cctgggttgcagttataccg-3'
Pla2g3	5'-ctgtgctgagggcaaagg-3'	5'-ctgaaatggagtcggctctt-3'
Pla2g4a	5'-gtgaggggctttattccaca-3'	5'-gaaacccccacctgaacc-3'
Pla2g4b	5'-cgcagctggtgagacttgt-3'	5'-cagccagctcctttggtc-3'
Pla2g4c	5'-cccacaacatatctaaggattaagg-3'	5'-acaggtggtgagtccactaatagat-3'
Pla2g4d	5'-gtcacccgagagactacatgg-3'	5'-acagtgagccggcagaag-3'
Pla2g4e	5'-gcaggatcccagacaaagc-3'	5'-ttgtcatcttccaggatggag-3'
Pla2g4f	5'-ggggcctcatcattgagtatt-3'	5'-cgtttcttgctggtctgaca-3'
Pla2g5	5'-ctcacactggcttggttcct-3'	5'-catggacttgagttctagcaagc-3'
Pla2g10	5'-cctctgtgacgggcaataat-3'	5'-atgtgacctcctggttgctt-3'
Pla2g12a	5'-gactgtgacgaggagttccag-3'	5'-gagctccaccgttgtctcac-3'
Pnpla1	5'-cgggtaccgggaagaaag-3'	5'-gccgtatttgaatcctcacc-3'
Pnpla2	5'-aatctctaccgcctctcgaa-3'	5'-ttccacacaggcctccag-3'
Pnpla3	5'-tgggagagctgtgctatcaa-3'	5'-gtggcccgttacagatgc-3'
Pnpla5	5'-acactgcccttcgagtatgc-3'	5'-ctcgggtagccactccatc-3'
Pnpla6	5'-aagccggcaccatcatag-3'	5'-tcaatcatgcgctggtacac-3'
Pnpla7	5'-ggagggggtggagctaga-3'	5'-cgccacactctgctagtgc-3'
Pnpla8	5'-ccaagctctctaatgttatcagca-3'	5'-aaggcaacaggccatcaa-3'
Pnpla9	5'-agggacgagaagcggagt-3'	5'-atgaccaggcctttcacg-3'
Tmem30a	5'-caacatccgtgagatcgaga-3'	5'-caagatgtcacattcggagataa-3'
Tmem30b	5'-cctgggcctcttctactcct-3'	5'-ggttgccggtgtagtcgta-3'
Tmem30c	5'-aactgtaccgccgactgaag-3'	5'-ccttggaacatggttactgga-3'
Xkr4	5'-ctgcgtcctgctcctttt-3'	5'-ccggctccggatacctaa-3'
Xkr8	5'-cctaaacagtgaggccaggta-3'	5'-gtataaaggtggcctgagcatc-3'
Xkr9	5'-gctgtttgagacctacctgga-3'	5'-aaaccatgatgaccatgcac-3'

	Taq man probe (Applied Bioscience) accession numbers		
Pla2g1b	Mm00478249_m1	*Pla2g2e*	Mm00478870_m1
Pla2g2c	Mm00476915_m1	*Pla2g2f*	Mm00478872_m1

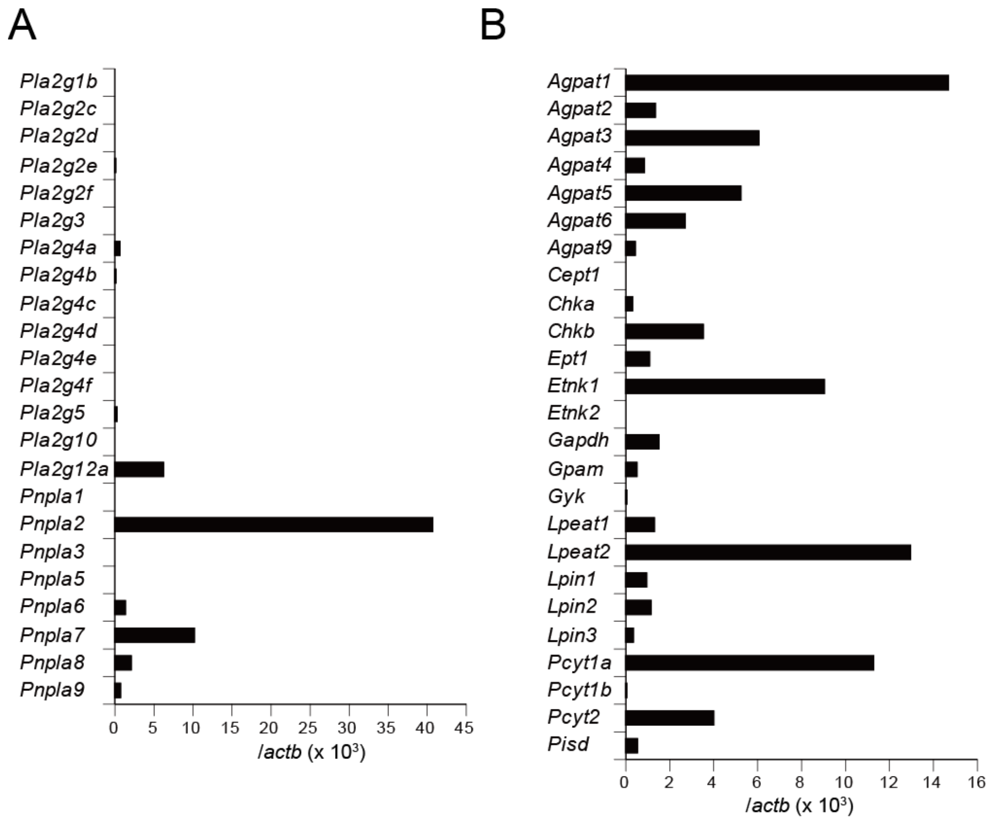

Supplementary Figure 1. Gene expressions of lipid-metabolic enzymes in osteoclasts. Osteoclast precursors were cultured with M-CSF and RANKL for 3 days. Total RNAs were isolated and quantitative PCR for lipid-degrading **(A)** and -biosynthetic **(B)** enzymes was performed with β-actin as a control.

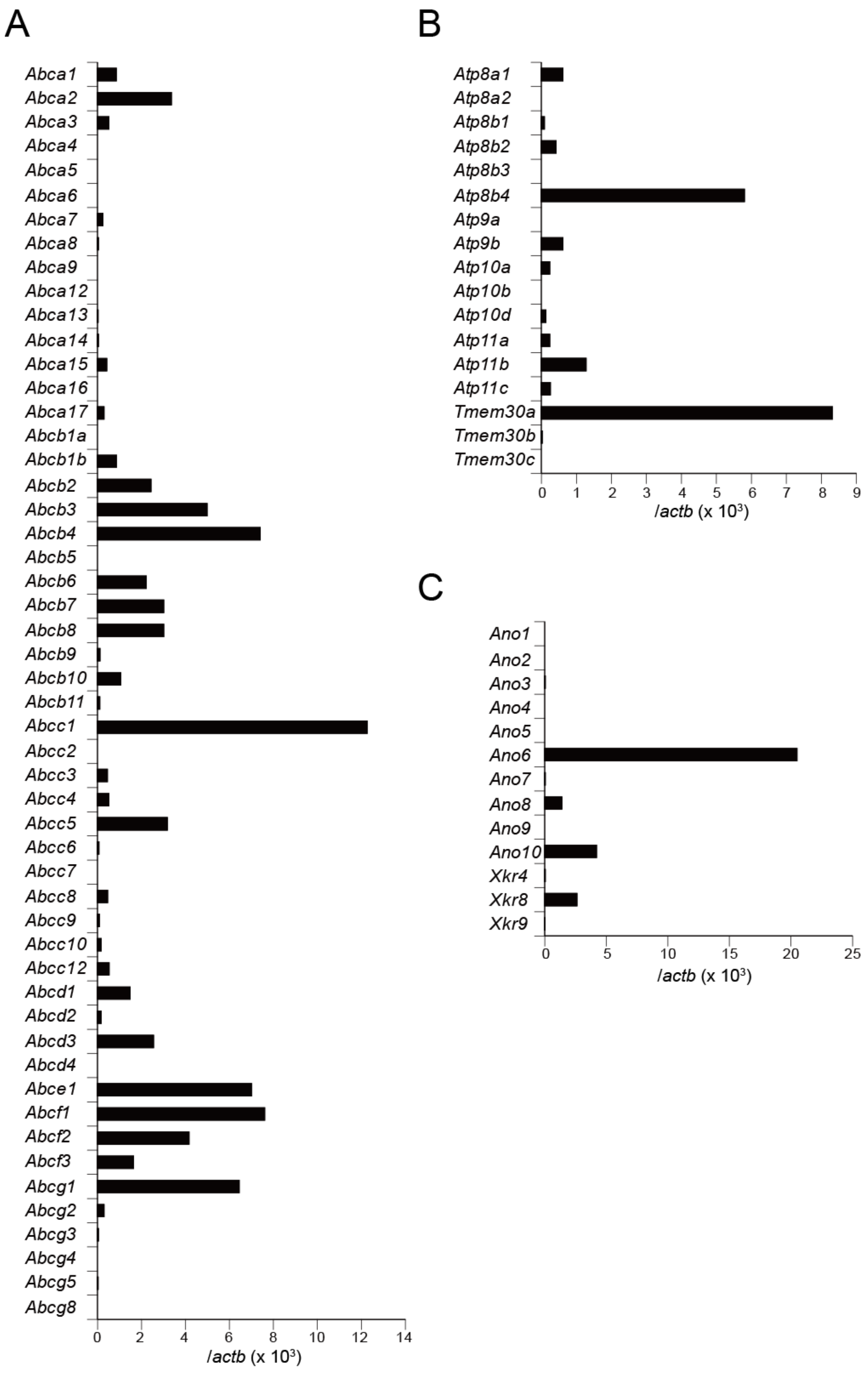

Supplementary Figure 2. Gene expressions of lipid transporters during osteoclastogenesis. Osteoclast precursors (day 0) were cultured with M-CSF and RANKL for 3 days. Total RNAs were isolated and quantitative PCR for ABC transporters (day 3) **(A)**, P-type ATPases (day 0) **(B)**, and anoctamins and Xkr proteins (day 3) **(C)** was performed with β-actin as a control. Note that the expressions of P-type ATPases on day 0 is shown in **(B)**, assuming that their reduced expression could be associated with the outward movement of PE.

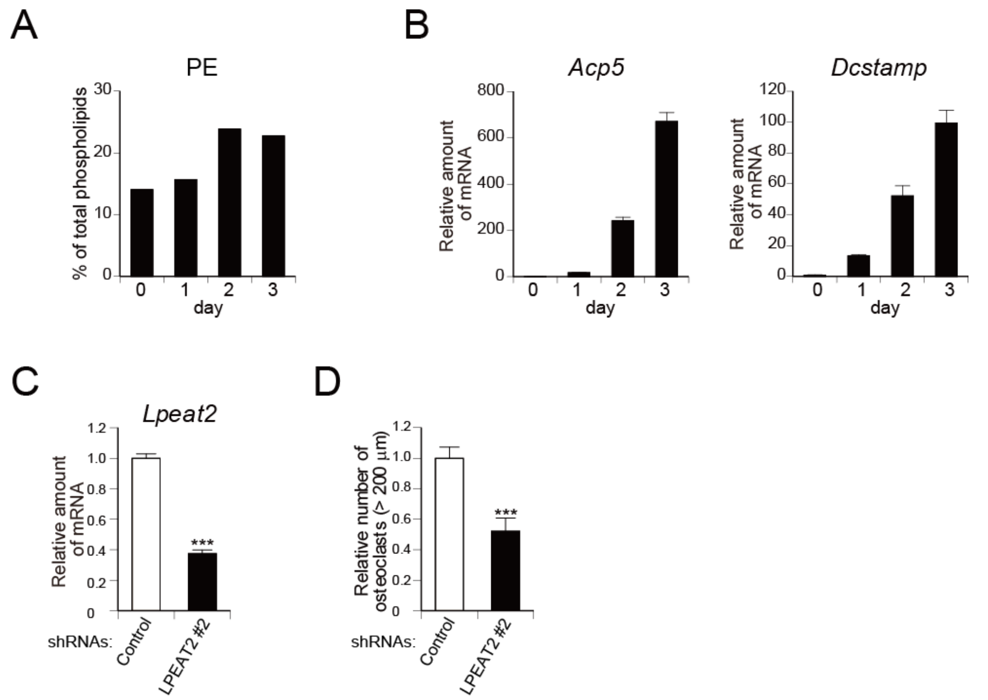

Supplementary Figure 3. Kinetic changes in osteoclast differentiation and shRNA-mediated knockdown of LPEAT2. (A) Kinetic changes in the ratio of PE during osteoclastogenesis. Osteoclast precursors (6 x 10^5 cells/well in 6-well plates) were cultured for the indicated periods with RANKL and M-CSF. Lipids were extracted from the cells, separated by two-dimensional thin layer chromatography, and quantified. Total phospholipid masses from the cells on day 0 to day 3 were 8.53, 12.74, 27.15 and 56.87 nmol/well, respectively. A representative result of two experiments is shown. **(B)** Kinetic changes in the expressions of osteoclast differentiation markers as assessed by quantitative PCR (n = 3). **(C and D)** Knockdown of LPEAT2. Expression levels of LPEAT2 (**C**) and osteoclast formation (**D**) after treatment with the second LPEAT2-directed shRNA are shown (n = 8). Each value is the mean ± SE.

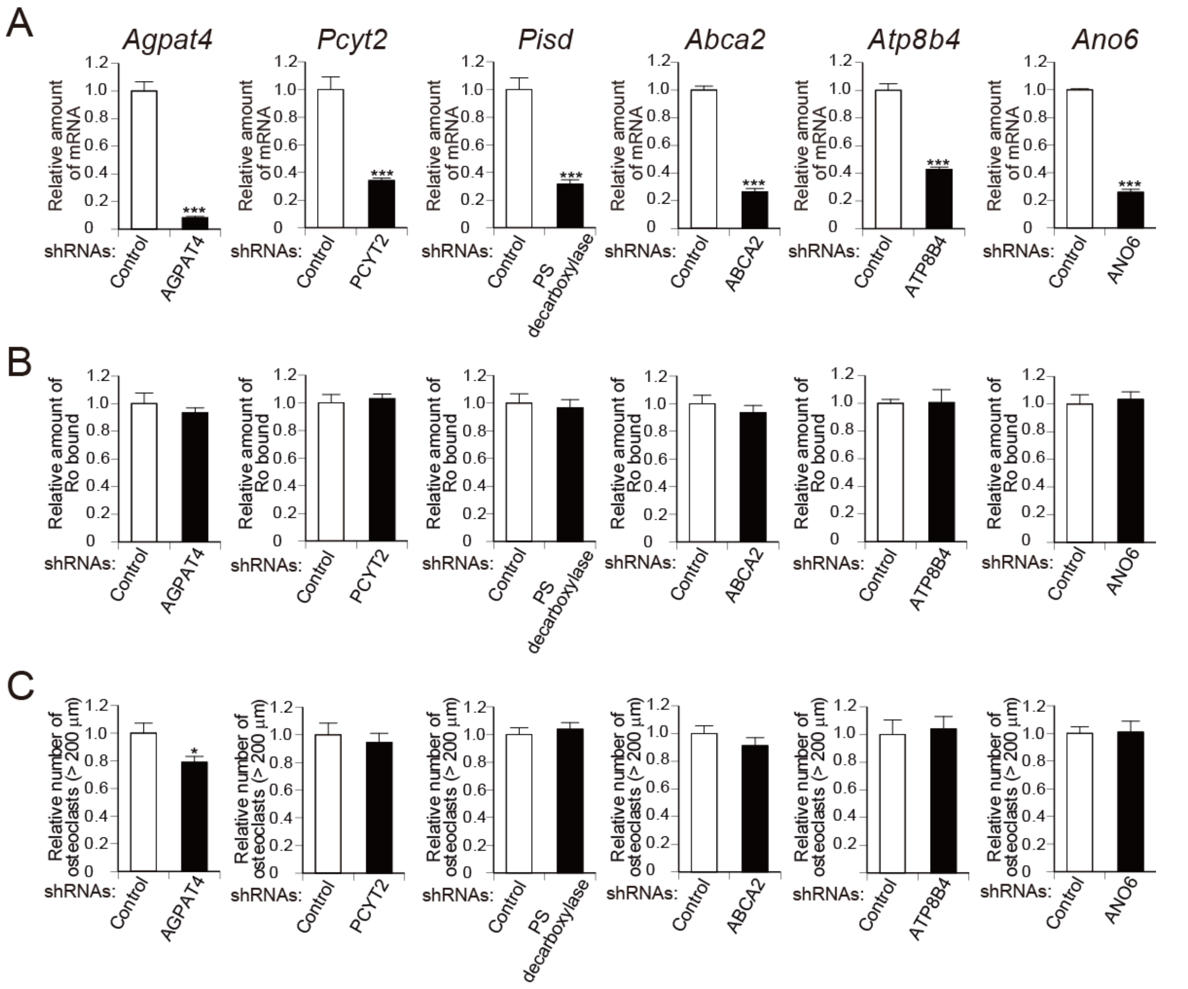

Supplementary Figure 4. shRNA-mediated knockdown of lipid-related molecules. Osteoclast precursors were infected with retroviruses expressing shRNA for the indicated genes or control, and then cultured with M-CSF and RANKL. **(A)** Efficiency of shRNA-mediated knockdown as analysed by quantitative PCR (n = 6 to 9). **(B)** The shRNA retrovirus-infected cells treated with SA-Bio-Ro were immunostained, and the fluorescence intensity was quantified (n = 4 to 8). **(C)** The shRNA retrovirus-infected cells were stained for TRAP and counted (n = 8). Each value is the mean ± SE.

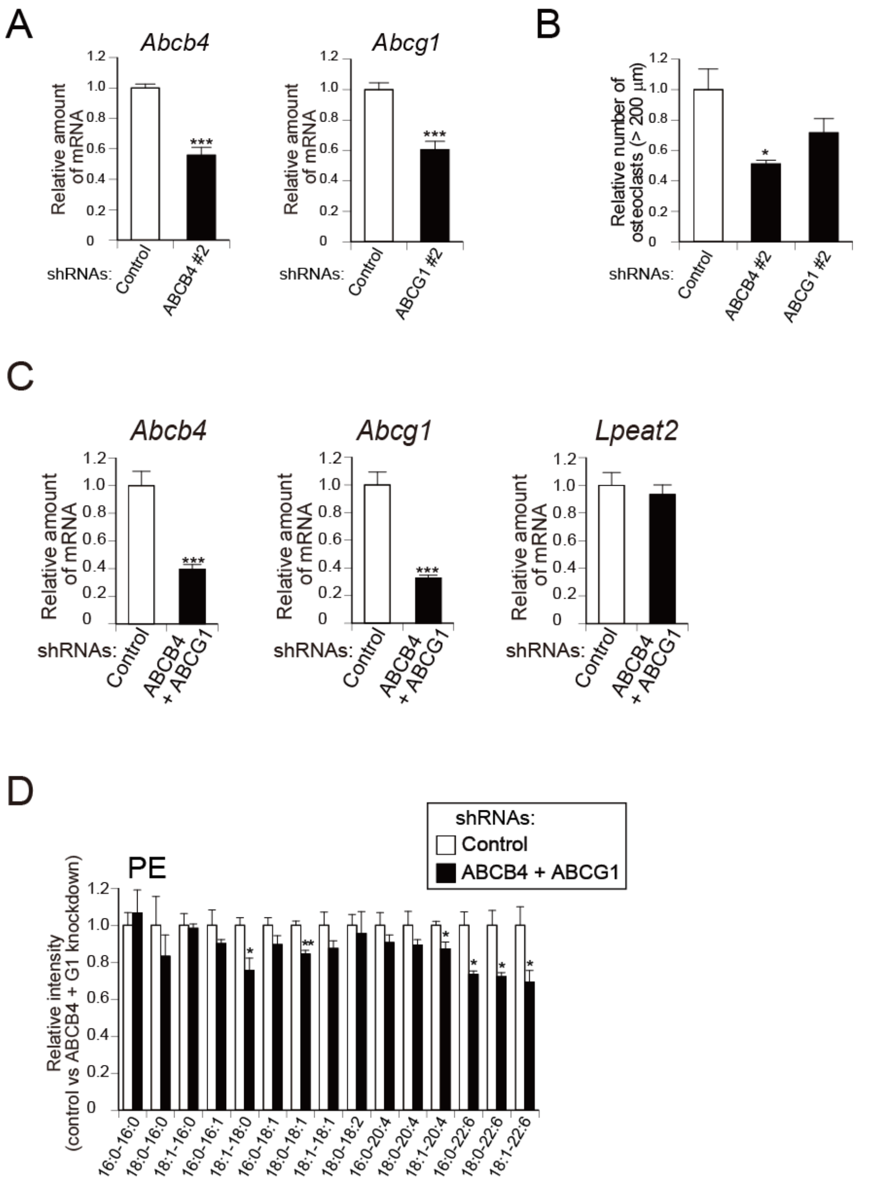

Supplementary Figure 5. Effects of shRNA-mediated knockdown of ABC transporters on osteoclast formation and PE content. (A and B) Knockdown of ABC transporters. Expression levels of ABCB4 and ABCG1 (**A**) and osteoclast formation (**B**) after treatment with the second shRNA for each are shown (n = 4). **(C and D)** The effect of double knockdown of ABCB4 and ABCG1 on PE content. **(C)** Expression levels of ABCB4, ABCG1 and LPEAT2 after treatment with ABCB4- and ABCG1-directed shRNAs are shown (n = 4). (**D**) The shRNA retrovirus-infected cells were subjected to MS analysis. Relative amounts (control *versus* ABCB4/ABCG1 knockdown, with control as 1) of individual PE species are quantified (n = 4). Each value is the mean ± SE.
